# 成人血液病患者接种新型冠状病毒疫苗中国专家共识（2022年版）

**DOI:** 10.3760/cma.j.issn.0253-2727.2022.05.002

**Published:** 2022-05

**Authors:** 中国康复医学会血液病康复专业委员会 中华医学会血液学分会

新型冠状病毒（以下简称“新冠病毒”）感染自2019年12月起在全球蔓延，2020年1月WHO将该病毒所致疾病命名为2019冠状病毒病（COVID-19），国际病毒分类学委员会将新冠病毒命名为重症急性呼吸综合征冠状病毒-2（SARS-CoV-2）。据WHO统计，截至2022年3月1日，全球累计确诊COVID-19病例超过4.3亿，死亡人数逾595万，其中我国累计确诊33.5万人，死亡0.62万人[1]。大规模接种新型冠状病毒疫苗（以下简称“新冠疫苗”），建立群体免疫屏障是有效防控新冠疫情、降低重症率和病死率的重要措施之一。随着新冠病毒不断演进，某些变异株的感染力和传播力增强，但研究表明新冠疫苗对其依然有较好的保护效力[2]，且疫苗的加强免疫还可进一步提高保护效力[3]–[4]。我国已在推进第三剂疫苗加强接种工作。截至2022年2月7日，全球疫苗接种率为55.52％，而我国接种率已达83.98％[1]。

与普通人群相比，血液病患者多数存在免疫功能受损，更易感染新冠病毒，且感染后重症率高，预后差。为了使这一特殊群体更好地通过接种疫苗获得最大可能的保护，避免成为群体免疫链条中的薄弱环节，我们充分梳理国内外现有文献，结合国家卫生健康委员会《新冠病毒疫苗接种技术指南（第一版）》[5]及国务院新冠疫情联防联控机制若干新闻发布会中专家的意见，参考国内外相关学术组织的推荐，对成人血液病患者接种新冠疫苗相关问题形成共识，提出以下建议，供同行参考。

一、血液病患者感染新冠病毒后预后不良，应强化常规防疫措施

建议：大多数血液病患者存在免疫功能受损，比普通人群感染SARS-CoV-2的风险更大，感染后重症率和病死率更高，因此，除需接种疫苗以外，其他各种常规感染防范措施应更加规范和严格。

血液病患者是新冠病毒感染后的重症、死亡高风险人群。武汉疫情时期血液肿瘤患者COVID-19发病率为10％，死亡率高达62％[6]（可能与疫情爆发初期人们对该病认识尚不足有关）。2020年初至2021年上半年，确诊COVID-19的血液肿瘤患者重症率高达44％，死亡率29％～43％，其中接受强化疗或免疫抑制剂治疗的患者死亡率较高，如成人急性淋巴细胞白血病（ALL）患者死亡率为33％，急性髓系白血病（AML）患者死亡率为43％，多发性骨髓瘤（MM）患者死亡率约33％，骨髓增殖性肿瘤（MPN）中骨髓纤维化患者死亡率达40％，且MPN患者感染新冠病毒后血栓发生率高达8.5％[7]–[11]。造血干细胞移植后确诊COVID-19的患者死亡率约21％，其中COVID-19相关死亡发生率在异基因造血干细胞移植患者中为20％，在自体造血干细胞移植患者中为14％[12]。血液肿瘤患者感染后死亡率是其他肿瘤患者的近2倍[13]–[14]。除血液病的疾病种类、疾病阶段和治疗状况外，老年患者、存在器官功能障碍、合并糖尿病、心脏病、高血压等慢性病也是影响生存的危险因素[7],[9],[15]。因此，进行新冠疫苗接种是血液病患者感染防控的重要举措。但研究表明，即使接种了疫苗的血液病患者仍有较高的突破性感染可能[16]，因而除疫苗接种以外，所有患者均应严格遵守国家和所在地规定的各项感染防控常规措施，如佩戴口罩、保持社交距离以及手卫生等，尤其要注重自我隔离，避免聚集。

二、血液病患者接种新冠疫苗的安全性

建议：因灭活疫苗工艺成熟，根据既往同类型疫苗的安全性特点，建议血液病患者优先选择；血液病患者免疫功能受损常见，合并其他慢性疾病者较多，接种疫苗后应加强不良反应的观察和处理，同时注意原发病的随访。

接种疫苗的不良反应，是指因疫苗本身特性引起的、与受种者个体差异有关的、与预防接种目的无关或者意外的反应，包括一般反应和异常反应。一般反应主要指由疫苗本身的特性引起的一过性反应，例如发热、局部红肿、硬结等症状，通常较轻微。异常反应主要指造成受种者的组织器官或功能损害的不良反应，罕见发生，如急性严重过敏反应等。新冠疫苗接种不良反应与已广泛应用的其他疫苗基本类似，目前我国批准附条件上市或紧急使用的新冠疫苗主要有三大类，分别为灭活疫苗、腺病毒载体疫苗和重组亚单位蛋白疫苗，不同的疫苗不良反应有所不同。据中国疾病预防控制中心信息，2020年12月15日至2021年4月30日，全国新冠疫苗接种2.65亿剂，报告不良反应发生率为11.86/10万剂次，其中一般反应占82.96％，如发热、红肿、硬结等。异常反应占17.04％，前三位有过敏性皮疹、血管性水肿、急性严重过敏反应，其中严重病例188例，报告发生率0.07/10万剂次。灭活疫苗在我国使用较多，Ⅲ期临床试验显示，接种后90％以上的不良反应为一般反应，无严重不良事件，其安全性与安慰剂对照组无显著差异[17]–[18]。mRNA疫苗在国外应用较广泛，常见不良反应与灭活疫苗相似，严重不良反应发生率为0.6％，无法耐受不良反应而中止疫苗接种的人群比例小于0.1％[19]。美国1700多万剂mRNA疫苗接种中出现过敏反应66例，无致死性病例，偶见mRNA疫苗接种后心肌炎或心包炎的发生[20]。

新冠疫苗接种后也可出现血液学相关不良反应，如免疫性血细胞减少[21]。但文献报道接种后免疫性血小板减少症（ITP）的发生率低于正常人群中原发ITP的发病率[22]。腺病毒载体疫苗接种后有发生免疫性血栓性血小板减少症的报道，发生率最高可达万分之一，有因此死亡的病例报道[23]。此外，还有疫苗接种后发生血液系统及其他免疫相关疾病的个案报道，例如，噬血细胞性淋巴组织细胞增多症（HLH）[24]–[25]、获得性血友病、自身免疫性溶血性贫血（AIHA）以及自身免疫性肝病、Guillain-Barré综合征等。对于血液病患者，目前文献中未检索到接种后出现与健康人群不同的严重不良事件报道。接种新冠疫苗是否会对原发的血液病产生影响，目前研究报道极少。意大利一项研究发现，新冠疫苗接种后有大约10％的AIHA和20％的ITP患者可观察到相应血常规指标下降，大多数使用糖皮质激素或调整原有治疗方案后可逐渐好转[26]，国内也有专家在临床工作中观察到此类病例。表明这类免疫功能紊乱的患者接种疫苗后有潜在的免疫相关疾病或原发病反复的风险，应在原发病及合并症稳定状态下，综合评估风险获益后谨慎考虑疫苗接种。

鉴于灭活疫苗的安全性特点较为明确，既往同类型疫苗较多，建议血液病患者优先选择，重组亚单位疫苗也可选用。此外，严重免疫功能受损的患者或异基因造血干细胞移植受者，一般禁止使用活疫苗（目前尚无新冠病毒减毒活疫苗）[27]。血液病患者接种疫苗后更应加强不良反应的观察，及时处理严重不良反应，同时注意对原发血液病的密切随访观察。对其他血小板减少和有出血倾向的患者（如血友病或抗凝治疗的患者）应注意疫苗接种注射部位的止血，有血栓形成倾向的患者（如原发性血小板增多症）应关注血小板计数和出凝血指标的变化。

三、血液病患者接种新冠疫苗后免疫应答有效性的影响因素

新冠疫苗接种后的免疫应答与患者的免疫功能状态密切相关：①免疫功能受损的血液病患者对新冠疫苗的免疫应答率和抗体水平总体低于健康人群；②治疗中的血液肿瘤患者免疫应答率和抗体水平低于未治疗或治疗完成且病情稳定的患者；③未缓解的血液肿瘤患者免疫应答率和抗体水平低于已缓解的患者；④强烈化疗、免疫抑制剂、针对B淋巴细胞的单克隆抗体药物或小分子靶向药物均可减弱体液和（或）细胞介导的免疫应答率和抗体水平；一般来说，治疗结束6～12个月后接种新冠疫苗免疫应答率和抗体水平明显增高；⑤自体造血干细胞移植3个月后、异基因造血干细胞移植或嵌合抗原受体T细胞（CAR-T细胞）治疗6个月后接种疫苗，免疫应答率和抗体水平随时间推移而逐渐提高；⑥新冠疫苗加强接种后可显著提高免疫应答率和抗体水平。

血液病疾病种类、疾病阶段、治疗方案、接种时机均可影响疫苗接种后机体的免疫应答水平。15％～25％的血液肿瘤患者接种新冠疫苗后机体无有效抗体产生，尤其是B细胞来源肿瘤，不论是否接受过抗B细胞治疗，抗体阳性率均较低[28]。以色列一组315例血液肿瘤患者接种mRNA疫苗后，免疫应答反应率最高的是霍奇金淋巴瘤和骨髓增生异常综合征（MDS），均达94％，最低的为慢性淋巴细胞白血病（CLL），为47％，其他依次为慢性髓性白血病（CML）、MPN、急性白血病、MM、侵袭性非霍奇金淋巴瘤（NHL）、惰性NHL，抗体滴度水平也依次递减[29]。非肿瘤性血液病患者接种新冠疫苗后免疫应答的情况报道极少。ITP、AIHA等免疫相关血细胞减少的患者及再生障碍性贫血（AA）患者自身存在免疫功能紊乱，且接受免疫抑制剂或中大剂量糖皮质激素等方案治疗期间免疫功能可能进一步受损，在停止治疗后的一定时间内，其对疫苗获得充分免疫应答的可能性也较低。

血液病治疗药物对新冠疫苗接种后免疫应答的影响也广受关注。如正在接受BTK抑制剂、Bcl-2抑制剂、抗CD20单抗治疗的患者，接种后抗体阳性率多在20％以下，停药后随时间的推移抗体阳性率逐步上升[30]–[33]，停药6～12个月后接种者抗体阳性率可达77％[34]。抗骨髓瘤治疗药物如抗CD38单抗、抗SLAMF7单抗、BCMA靶向药物等也会通过影响机体免疫微环境，降低疫苗接种效果[35]–[38]。但有研究表明，使用来那度胺不是新冠病毒感染死亡的危险因素，而且还可能用于COVID-19重症患者的治疗，因此国外有研究推荐来那度胺单药维持治疗的MM患者可以接种新冠疫苗[39]–[40]。有两项前瞻性研究提示，针对BCR-ABL的酪氨酸激酶抑制剂（TKI）治疗不影响新冠疫苗的免疫应答，且TKI治疗的CML患者罹患COVID-19后，体内中和抗体生成水平与普通人群相似，ICU入住率和机械通气率反有降低趋势[41]–[42]，应用TKI治疗的Ph阳性ALL患者COVID-19的发病率也相对较低[43]。

疾病的不同阶段和接种时机也影响免疫应答水平。原发病控制稳定或治疗结束较长时间的血液肿瘤患者，接种疫苗后免疫应答较好，一组AML/MDS患者接种两剂疫苗后抗体阳性率可达95.7％，其中87％的患者接种时处于疾病缓解状态[44]。初诊未治疗的CLL进行疫苗接种，抗体阳性率为61％，治疗结束后持续临床缓解状态时接种，抗体阳性率可达85％以上[30]–[32]；MM患者在疾病进展期接种者，抗体阳性率为30％，而达到部分缓解及以上者抗体阳性率则大于63％，且MM患者抗体阳性率（53.5％）低于冒烟型骨髓瘤（60.5％）和意义未明单克隆丙种球蛋白病患者（84％）[35]–[36]；异基因造血干细胞移植6个月后且无急性移植物抗宿主病（GVHD）的一组患者接种疫苗后，抗体阳性率为77.6％，慢性GVHD及无免疫抑制治疗的患者中和抗体水平较高[45]；而CAR-T细胞治疗后免疫应答研究的病例数较少。有研究提示，外周血淋巴细胞计数<1×10^9^/L、3个月内接受过免疫抑制治疗的患者接种疫苗后抗体滴度水平偏低[46]。

新冠疫苗加强接种可提高抗体阳性率及抗体水平。健康人群第三针灭活疫苗接种后28 d，中和抗体水平可提高3～5倍[47]。对疫苗接种后抗体阳性率较低的B细胞肿瘤患者行加强接种后，65％的患者体内中和抗体水平提升，对接种后抗体阴性的CLL患者加强接种后，抗体阳性率可达23.8％[48]–[49]。对接种两剂疫苗后抗体水平低于保护阈值的造血干细胞移植患者行加强接种后，抗体滴度也明显增高，近一半患者抗体水平可达到或超过保护阈值[50]。

四、对血液病患者接种新冠疫苗的建议

血液病患者对新冠疫苗的免疫应答总体低于健康人群，但多数情况下，疫苗接种产生的免疫应答对患者具有保护效力。在不影响原发病治疗、充分权衡风险效益、患者自愿和知情同意的前提下，应鼓励符合接种条件且无禁忌证的血液病患者接种疫苗，同时加强不良反应监测及原发病的随访。血液病患者的家人、照护者及其他密切接触者，如无接种禁忌，均应接种疫苗。

1. 接种建议：

（1）应接种的患者：完成治疗1年以上且处于疾病完全缓解状态、病情稳定的血液肿瘤患者；与免疫紊乱或凝血障碍无关的非恶性血液病、且病情已控制的患者，如缺铁性贫血、巨幼细胞性贫血患者。

（2）可接种的患者：完成治疗3个月以上、病情稳定的血液肿瘤患者；自体造血干细胞移植3个月以上、异基因造血干细胞移植和细胞免疫治疗（如CAR-T）结束6个月以上, 且中性粒细胞和淋巴细胞计数恢复、原发病稳定的患者[51]；移植前或CAR-T细胞治疗前接种过疫苗的患者，移植或CAR-T细胞治疗6个月后需适时重新接种疫苗；应用来那度胺或针对BCR-ABL的TKI单药维持治疗且病情控制的患者。

（3）可考虑优化疫苗接种时间的患者：未治疗的新发惰性血液肿瘤患者，在病情允许的情况下，可考虑先接种疫苗再启动抗肿瘤治疗。若血液肿瘤患者在治疗间歇期接种，应处于疾病稳定状态，且外周血中性粒细胞计数≥1.0×10^9^/L及血小板计数≥50×10^9^/L。

（4）既往确诊过COVID-19的患者，治愈出院6个月后可接种单剂疫苗[52]–[54]。

（5）符合接种条件的血液病患者，均应适时进行疫苗加强接种。

（6）正在参加临床试验的患者，可与发起单位或牵头专家讨论疫苗接种事宜[55]。

2. 建议不接种或暂缓接种的患者：

（1）处于疾病进展期、病情未有效控制的患者；

（2）正在接受抗B细胞治疗如CD20单抗、CD19/CD3等双靶向药物或BTK抑制剂维持治疗或结束治疗不满6个月的患者[30]–[32]；

（3）强化疗或免疫抑制剂（如ATG、阿伦单抗、钙调抑制剂、中大剂量糖皮质激素等）治疗中的患者；

（4）中性粒细胞计数<1.0×10^9^/L、血小板计数<50×10^9^/L的患者[52]–[53]；

（5）免疫相关血细胞减少性疾病，如ITP、AIHA、AA等，病情未控制者不宜接种新冠疫苗，停用免疫抑制剂未满6个月的患者，接种新冠疫苗应谨慎；

（6）既往有HLH病史的患者或EB病毒活动性感染相关的血液病患者；

（7）有血栓性血小板减少性紫癜（TTP）病史或肥大细胞增多症病史的患者；

（8）血液病相关凝血功能障碍的患者；

（9）自体造血干细胞移植3个月内、异基因造血干细胞移植或CAR-T细胞治疗6个月内，或存在Ⅱ～Ⅳ度或难治性急性GVHD或广泛性慢性GVHD的患者；

（10）存在国家卫生健康委员会《新冠病毒疫苗接种技术指南（第一版）》[5]描述的疫苗接种禁忌证的患者，例如，有疫苗或其成分过敏史，合并妊娠，合并严重慢性疾病或慢性病急性发作，合并神经精神疾病，正在发热，合并急性感染或合并急性心、肺、肝、肾功能异常的患者。

五、总结

综上，本共识复习了COVID-19疫情暴发以来的相关文献、相关学术组织的推荐意见和国家权威部门发布的指南或专家建议，综合了共识编撰专家的临床经验和意见，以供血液病工作者及相关专业的医务人员参考。给出的建议考虑了疫苗潜在的不良反应，对原发血液病及其治疗的可能影响及现有的免疫应答有效性数据，实施时需将接种、暂缓接种或不接种的所有建议综合考虑，密切结合患者的具体情况和疫情风险，充分评估接种疫苗的风险/获益。所引用的文献多数为回顾性观察，对不同的血液系统疾病的全面、高质量的循证医学证据尚待积累。随着血液病患者新冠疫苗接种相关数据的不断增加，本共识将会依据新的临床证据和国家防疫策略进行相应调整和修订。

## References

[1] World Health Organization Coronavirus disease (COVID-19) pandemic[S].

[2] Li XN, Huang Y, Wang W (2021). Effectiveness of inactivated SARS-CoV-2 vaccines against the Delta variant infection in Guangzhou: a test-negative case-control real-world study[J]. Emerg Microbes Infect.

[3] Wang K, Cao YL, Zhou YJ (2021). A third dose of inactivated vaccine augments the potency, breadth, and duration of anamnestic responses against SARS-CoV-2[J]. medRxiv.

[4] Zhao X, Li D, Ruan W (2022). Effects of a Prolonged Booster Interval on Neutralization of Omicron Variant[J]. N Engl J Med.

[5] 国家卫生健康委员会疾病预防控制局 (2021). 新冠病毒疫苗接种技术指南(第一版)[J]. 中国病毒病杂志.

[6] He W, Chen L, Chen L (2020). COVID-19 in persons with haematological cancers[J]. Leukemia.

[7] Ribera JM, Morgades M, Coll R (2021). Frequency, Clinical Characteristics and Outcome of Adults With Acute Lymphoblastic Leukemia and COVID 19 Infection in the First vs. Second Pandemic Wave in Spain[J]. Clin Lymphoma Myeloma Leuk.

[8] Palanques-Pastor T, Megías-Vericat JE, Martínez P (2021). Characteristics, clinical outcomes, and risk factors of SARS-COV-2 infection in adult acute myeloid leukemia patients: experience of the PETHEMA group[J]. Leuk Lymphoma.

[9] Chari A, Samur MK, Martinez-Lopez J (2020). Clinical features associated with COVID-19 outcome in multiple myeloma: first results from the International Myeloma Society data set[J]. Blood.

[10] Barbui T, Vannucchi AM, Alvarez-Larran A (2021). High mortality rate in COVID-19 patients with myeloproliferative neoplasms after abrupt withdrawal of ruxolitinib[J]. Leukemia.

[11] Barbui T, De Stefano V, Alvarez-Larran A (2021). Among classic myeloproliferative neoplasms, essential thrombocythemia is associated with the greatest risk of venous thromboembolism during COVID-19[J]. Blood Cancer J.

[12] Sharma A, Bhatt NS, St Martin A (2021). Clinical characteristics and outcomes of COVID-19 in haematopoietic stem-cell transplantation recipients: an observational cohort study[J]. Lancet Haematol.

[13] Fu C, Stoeckle JH, Masri L (2021). COVID-19 outcomes in hospitalized patients with active cancer: Experiences from a major New York City health care system[J]. Cancer.

[14] de Joode K, Tol J, Hamberg P (2022). Life-prolonging treatment restrictions and outcomes in patients with cancer and COVID-19: an update from the Dutch Oncology COVID-19 Consortium[J]. Eur J Cancer.

[15] Vijenthira A, Gong IY, Fox TA (2020). Outcomes of patients with hematologic malignancies and COVID-19: a systematic review and meta-analysis of 3377 patients[J]. Blood.

[16] Wang L, Kaelber DC, Xu R (2022). COVID-19 breakthrough infections, hospitalizations and mortality in fully vaccinated patients with hematologic malignancies: A clarion call for maintaining mitigation and ramping-up research[J]. Blood Rev.

[17] Tanriover MD, Doğanay HL, Akova M (2021). Efficacy and safety of an inactivated whole-virion SARS-CoV-2 vaccine (CoronaVac): interim results of a double-blind, randomised, placebo-controlled, phase 3 trial in Turkey[J]. Lancet.

[18] Ella R, Reddy S, Blackwelder W (2021). Efficacy, safety, and lot-to-lot immunogenicity of an inactivated SARS-CoV-2 vaccine (BBV152): interim results of a randomised, double-blind, controlled, phase 3 trial[J]. Lancet.

[19] Baden LR, El Sahly HM, Essink B (2021). Efficacy and Safety of the mRNA-1273 SARS-CoV-2 Vaccine[J]. N Engl J Med.

[20] Shimabukuro TT, Cole M, Su JR (2021). Reports of Anaphylaxis After Receipt of mRNA COVID-19 Vaccines in the US-December 14, 2020-January 18, 2021[J]. JAMA.

[21] Welsh KJ, Baumblatt J, Chege W (2021). Thrombocytopenia including immune thrombocytopenia after receipt of mRNA COVID-19 vaccines reported to the Vaccine Adverse Event Reporting System (VAERS)[J]. Vaccine.

[22] Simpson CR, Shi T, Vasileiou E (2021). First-dose ChAdOx1 and BNT162b2 COVID-19 vaccines and thrombocytopenic, thromboembolic and hemorrhagic events in Scotland[J]. Nat Med.

[23] Tregoning JS, Flight KE, Higham SL (2021). Progress of the COVID-19 vaccine effort: viruses, vaccines and variants versus efficacy, effectiveness and escape[J]. Nat Rev Immunol.

[24] Tang LV, Hu Y (2021). Hemophagocytic lymphohistiocytosis after COVID-19 vaccination[J]. J Hematol Oncol.

[25] Baek DW, Hwang S, Kim J (2022). Patients presenting high fever with lymphadenopathy after COVID-19 vaccination were diagnosed with hemophagocytic lymphohistiocytosis[J]. Infect Dis (Lond).

[26] Fattizzo B, Giannotta JA, Cecchi N (2021). SARS-CoV-2 vaccination in patients with autoimmune cytopenias: The experience of a reference center[J]. Am J Hematol.

[27] Piñana JL, Vázquez L, Martino R (2022). Spanish Society of Hematology and Hemotherapy expert consensus opinion for SARS-CoV-2 vaccination in onco-hematological patients[J]. Leuk Lymphoma.

[28] Greenberger LM, Saltzman LA, Senefeld JW (2021). Antibody response to SARS-CoV-2 vaccines in patients with hematologic malignancies[J]. Cancer Cell.

[29] Herzog Tzarfati K, Gutwein O, Apel A (2021). BNT162b2 COVID-19 vaccine is significantly less effective in patients with hematologic malignancies[J]. Am J Hematol.

[30] Benjamini O, Rokach L, Itchaki G (2022). Safety and efficacy of the BNT162b mRNA COVID-19 vaccine in patients with chronic lymphocytic leukemia[J]. Haematologica.

[31] Roeker LE, Knorr DA, Thompson MC (2021). COVID-19 vaccine efficacy in patients with chronic lymphocytic leukemia[J]. Leukemia.

[32] Molica S, Giannarelli D, Lentini M (2021). Efficacy of the BNT162b2 mRNA COVID-19 Vaccine in Patients with Chronic Lymphocytic Leukemia: A Serologic and Cellular Study[J]. Chemotherapy.

[33] Morawska M (2022). Reasons and consequences of COVID-19 vaccine failure in patients with chronic lymphocytic leukemia[J]. Eur J Haematol.

[34] Vijenthira A, Gong I, Betschel SD (2021). Vaccine response following anti-CD20 therapy: a systematic review and meta-analysis of 905 patients[J]. Blood Adv.

[35] Ludwig H, Boccadoro M, Moreau P (2021). Recommendations for vaccination in multiple myeloma: a consensus of the European Myeloma Network[J]. Leukemia.

[36] Bird S, Panopoulou A, Shea RL (2021). Response to first vaccination against SARS-CoV-2 in patients with multiple myeloma[J]. Lancet Haematol.

[37] Terpos E, Gavriatopoulou M, Ntanasis-Stathopoulos I (2021). The neutralizing antibody response post COVID-19 vaccination in patients with myeloma is highly dependent on the type of anti-myeloma treatment[J]. Blood Cancer J.

[38] Henriquez S, Zerbit J, Bruel T (2022). Anti-CD38 therapy impairs SARS-CoV-2 vaccine response against alpha and delta variants in patients with multiple myeloma[J]. Blood.

[39] Krejci M, Pour L, Adam Z (2021). Outcome of COVID-19 infection in 50 multiple myeloma patients treated with novel drugs: single-center experience[J]. Ann Hematol.

[40] Sundaresan L, Giri S, Singh H (2021). Repurposing of thalidomide and its derivatives for the treatment of SARS-coV-2 infections: Hints on molecular action[J]. Br J Clin Pharmacol.

[41] Claudiani S, Apperley JF, Parker EL (2022). Durable humoral responses after the second anti-SARS-CoV-2 vaccine dose in chronic myeloid leukaemia patients on tyrosine kinase inhibitors[J]. Br J Haematol.

[42] Bonifacio M, Tiribelli M, Miggiano MC (2021). The serological prevalence of SARS-CoV-2 infection in patients with chronic myeloid leukemia is similar to that in the general population[J]. Cancer Med.

[43] Foà R, Bonifacio M, Chiaretti S (2020). Philadelphia-positive acute lymphoblastic leukaemia (ALL) in Italy during the COVID-19 pandemic: a Campus ALL study[J]. Br J Haematol.

[44] Jain AG, Dong NC, Ball S (2021). Responses to Sars-Cov-2 Vaccines in Patients with Myelodysplastic Syndrome and Acute Myeloid Leukemia[J]. Blood.

[45] Shem-Tov N, Yerushalmi R, Danylesko I (2022). Immunogenicity and safety of the BNT162b2 mRNA COVID-19 vaccine in haematopoietic stem cell transplantation recipients[J]. Br J Haematol.

[46] Redjoul R, Le Bouter A, Beckerich F (2021). Antibody response after second BNT162b2 dose in allogeneic HSCT recipients[J]. Lancet.

[47] Zeng G, Wu Q, Pan H (2022). Immunogenicity and safety of a third dose of CoronaVac, and immune persistence of a two-dose schedule, in healthy adults: interim results from two single-centre, double-blind, randomised, placebo-controlled phase 2 clinical trials[J]. Lancet Infect Dis.

[48] Greenberger LM, Saltzman LA, Senefeld JW (2021). Anti-spike antibody response to SARS-CoV-2 booster vaccination in patients with B cell-derived hematologic malignancies[J]. Cancer Cell.

[49] Herishanu Y, Rahav G, Levi S (2022). Efficacy of a third BNT162b2 mRNA COVID-19 vaccine dose in patients with CLL who failed standard 2-dose vaccination[J]. Blood.

[50] Redjoul R, Le Bouter A, Parinet V (2021). Antibody response after third BNT162b2 dose in recipients of allogeneic HSCT[J]. Lancet Haematol.

[51] European Society for Blood and Marrow Transplantation CORONAVIRUS DISEASE COVID-19: EBMT RECOMMENDATIONS VERSION 17 - January 26, 2022.

[52] NCCN: Cancer and COVID-19 Vaccination. Version 5.0 01/04/2022.

[53] NCCN COVID-19 Vaccination Guide for People With Cancer.

[54] Ludwig H, Sonneveld P, Facon T (2021). COVID-19 vaccination in patients with multiple myeloma: a consensus of the European Myeloma Network[J]. Lancet Haematol.

[55] Desai A, Gainor JF, Hegde A (2021). COVID-19 vaccine guidance for patients with cancer participating in oncology clinical trials[J]. Nat Rev Clin Oncol.

